# Supplementary material for: The genome sequence of the Mountain Clouded Yellow, Colias phicomone (Esper, 1780) (Lepidoptera: Pieridae)
Source: Wellcome Open Res. 2025 Jul 28;10:379. [Version 1] doi: 10.12688/wellcomeopenres.24625.1 (PMC12451261; doi:10.12688/wellcomeopenres.24625.1)
Supplement: Peer review history

# Reviewer report: Reviewer response for version 1

**DOI:** 10.21956/wellcomeopenres.27136.r129072

**Published:** 2025-09-22

**Authors:** Marco Pessoa-Filho

**Affiliations:** 1 Brazilian Agricultural Research Corporation, Brasília, Brazil

The manuscript describes the sequencing and assembly of a chromosome-scale, haplotype-phased genome for *Colias phicomone*. The authors used PacBio HiFi sequences and Hi-C library sequencing to achieve this. Methods for library construction and sequencing are clearly described. Methods for draft assembly and scaffolding are presented. Manual adjustments were performed to the Hi-C assembly. Quality metrics are included.

Are sufficient details of methods and materials provided to allow replication by others?

Yes

Is the rationale for creating the dataset(s) clearly described?

Yes

Are the datasets clearly presented in a useable and accessible format?

Yes

Are the protocols appropriate and is the work technically sound?

Yes

Reviewer Expertise:

Plant genomics, bioinformatics, genome assembly and annotation, variant discovery

I confirm that I have read this submission and believe that I have an appropriate level of expertise to confirm that it is of an acceptable scientific standard.

# Reviewer report: Reviewer response for version 1

**DOI:** 10.21956/wellcomeopenres.27136.r129069

**Published:** 2025-09-04

**Authors:** Irene Julca

**Affiliations:** 1 University of Lausanne, Lausanne, Switzerland

**Linke and colleagues present the chromosome-level, haplotype-resolved genome assembly of a female Colias phicomone specimen, along with the assembly of its mitochondrial genome. The manuscript is clearly written and well presented, and the reported genome represents a valuable resource for the scientific community. My only suggestion concerns Figure 2: the authors could provide additional information in the main text about the observed pattern of the Z1 and Z2 chromosomes when referencing this figure.**

Are sufficient details of methods and materials provided to allow replication by others?

Yes

Is the rationale for creating the dataset(s) clearly described?

Yes

Are the datasets clearly presented in a useable and accessible format?

Yes

Are the protocols appropriate and is the work technically sound?

Yes

Reviewer Expertise:

Genomics, comparative genomics, phylogenomics, transcriptomics

I confirm that I have read this submission and believe that I have an appropriate level of expertise to confirm that it is of an acceptable scientific standard.
